# Neoadjuvant therapy in pancreatic neuroendocrine neoplasms: A systematic review and meta-analysis

**DOI:** 10.3389/fonc.2022.981575

**Published:** 2022-11-24

**Authors:** Yongzheng Li, Zhiyao Fan, Feifei Zhang, Jian Yang, Ming Shi, Shujie Liu, Yufan Meng, Hanxiang Zhan

**Affiliations:** ^1^ Division of Pancreatic Surgery, Department of General Surgery, Qilu Hospital, Shandong University, Jinan, Shandong, China; ^2^ The First Operating Theater, Qilu Hospital, Shandong University, Jinan, Shandong, China

**Keywords:** pancreatic neuroendocrine tumor, neoadjuvant therapy, surgery, tumor response, prognosis

## Abstract

**Background and Objectives:**

Neoadjuvant therapy plays an increasingly important role in pancreatic neuroendocrine neoplasms (pNENs), but the systematic evaluation of its efficacy is still lacking. The purpose of this study is to explore the role of neoadjuvant therapy in pNENs.

**Methods:**

We systematically reviewed the literatures published online until October 1, 2021. Meta-analysis was conducted to generate proportion with 95% confidence intervals (95% CI) for tumor response, resection rate, R0 resection rate and survival time.

**Results:**

Nine studies with 468 patients were involved in the systematic review. None of these patients met complete response (CR). Furthermore, 43.6% (95% CI [18.1, 69.0]) patients were expected to achieve partial response (PR), 51.3% (95% CI [27.9, 78.3]) to stable disease (SD), and 4.3% (95% CI [0.7, 7.9]) to progressive disease (PD). The estimate resection rate and R0 resection rate after neoadjuvant therapy were 68.2% (95% CI [44.5, 91.9]) and 60.2% (95% CI [53.5, 66.9]), respectively. There was no significant difference in resection rate between different chemotherapy regimens (41.67% vs 33.93%, P=0.453), as well as R0 resection rate (62.50% vs 68.30%, P=0.605). In terms of objective response rate (ORR), there was no significant difference between CAPTEM and FAS (41.67% vs 33.93%, P=0.453), while PRRT showed a higher ORR compared with chemotherapy, although there was also no statistical difference (49.06% vs 36.96%, P=0.154).

**Conclusion:**

Neoadjuvant therapies could reduce the tumor size and stage of some borderline resectable or unresectable pNENs, and give some patients the chance of radical resection. However, according to the current data, the best treatment regimen for pNENs neoadjuvant therapy is still unknown.

## 1 Introduction

Pancreatic neuroendocrine neoplasms (pNENs) are rare diseases. It originated from pancreatic neuroendocrine cells and only accounted for 1%-2% of pancreatic neoplasms (1, 2). However, due to the continuous improvement of clinicians’ understanding of this disease and widespread use of cross-sectional imaging, the incidence or detection rate of gastroenteropancreatic neuroendocrine neoplasm (GEP-NENs) increased from 1.09 per 100,000 to 6.98 per 100,000, between 1973 and 2012, a nearly seven-fold increase, according to the Surveillance, Epidemiology, and End Results (SEER) program of the National Cancer Institute, and pNENs accounted for about 12% of those cases (3, 4).

Based on the differentiation degree and Ki-67 index of the tumor, as early as 2010, the World Health Organization (WHO) classified neuroendocrine neoplasms into well-differentiated neuroendocrine tumors (NET) G1 stage, G2 stage and poorly differentiated neuroendocrine carcinoma (NEC) (5). With the continuous progress of the understanding of NENs, in 2017, the WHO classified the well differentiated portion of the original NEC family as neuroendocrine tumor G3 and the poorly differentiated group as NEC (6). And this classification was first applied to the pancreas. Meanwhile, pNENs were highly heterogeneous diseases with various components, including functional pNENs (such as insulinoma, gastrinoma, glucagonoma, etc.) and non-functional pNENs which account for about 70%-75% (2, 3, 7). Due to its high heterogeneity, the treatment options for pNENs are diverse. Currently, the main treatment methods include surgical resection, peptide receptor radionuclide therapy (PRRT) and medicine therapy (mainly including somatostatin, targeted drugs and chemotherapy, etc.) (8). Although the treatment of G3 NETs and NECs are different from G1 NETs and G2 NETs, radical surgical treatment is the only possible method to cure pNENs (9–12). However, as mentioned above, due to the high proportion of non-functional pNENs, patients have no obvious clinical symptoms in the early stage, only about 38% patients found pNENs incidentally by cross sectional imaging (7). Most patients are treated only when tumor compressed or invaded the surrounding organs, at that time, about 60%-70% of patients had local advanced diseases or distant metastasis, and surgical treatment alone was of limited benefit or was even inoperable (13, 14). Hence, multidisciplinary therapy played an increasingly important role in the treatment of pNENs with the aim of maximizing the patient’s benefits.

For patients with locally advanced disease or distant metastasis, surgical therapy or interventional therapy could prolong the survival time of patients with pNENs, but some studies had shown that patients underwent chemotherapy in addition to surgical resection were superior to surgical treatment only (10, 15). Therefore, the neoadjuvant therapy for pNENs had made great progress. Its objective was to reduce tumor load and tumor stage, so that patients who were previously unable to receive surgical treatment could get the chance of surgical resection again, and at the same time reduced the risk of tumor recurrence. Although many studies had shown the positive effects of neoadjuvant therapy, such as chemotherapy, PRRT and targeted therapy, in tumor reduction, successful transformation into surgical resection and prolonging the survival time of patients, there was still a lack of systematic evaluation of the efficacy of neoadjuvant therapy (16–18). In our study, we systematically reviewed related studies on neoadjuvant therapy for pNENs and conducted a meta-analysis, aiming to explore the tumor response, surgical resection rate, R0 resection rate and the long-term survival after neoadjuvant therapy.

## 2 Materials and methods

Our study follows the PRISMA (preferred reporting items for systematic reviews and meta-analyses) reporting guidelines and the checklist is showed in **Supplementary Table 1**. The protocol of the current study was developed and previously registered on PROSPERO (registration ID: CRD42021284146).

### 2.1 Search strategy and study selection criteria

We systematically searched the literature published in the Web of Science, Pubmed, Embase and Cochrane Library database. And the deadline for retrieval was set to October 1, 2021. We screened the studies based on the keywords: “pancreatic neuroendocrine/endocrine/islet” and “tumors/neoplasm/cancer” and “neoadjuvant/preoperative” and “therapy/chemotherapy/systemic therapy/radiation”. And the publications in reference section and the previous relevant meta-analysis and reviews were further searched to avoid omissions. Two researchers (Y.Z. Li and Z.Y. Fan) independently screened the relevant literatures. The title and abstract of per study were preliminarily screened to exclude the unqualified studies. Then, the full text of the remaining studies was further reviewed to obtain the studies included in the current study. The inclusion and exclusion criteria are as follows. Inclusion criteria: (1) studies of neoadjuvant therapy for pancreatic neuroendocrine neoplasms; (2) studies that revealed the relevant efficacy information or prognostic information after therapy, such as tumor response, surgical rate survival state and so on. Exclusion criteria: (1) publications which included the same study patients; (2) the type of study is case report, review, meta-analysis, letter to the editor, conference record; (3) duplicate studies; (4) key data for outcomes was missing. The NOS (Newcastle-Ottawa Scale) was used to assess the quality of the included studies (**Supplementary Table 2**).

### 2.2 Data processing

Two researchers (J. Yang and M. Shi) extracted the data of the included studies independently. The final results were integrated, and the differences were negotiated with the third author (H.X. Zhan). Finally, the baseline data and clinical information, such as the first author, published year, country, age, tumor types, tumor response, median follow-up and so on, were got from selected studies.

In the current study, the primary outcomes were tumor response and resectability, and the secondary outcomes were histopathological changes, complication rate, survival time. Tumor response was accessed by the Response Evaluation Criteria in Solid Tumors (RECIST) version 1.1 (19): (1) Complete response (CR): the lesion disappeared on imaging or the short diameter of all pathological lymph nodes (including target and non-target nodes) must be reduced to < 10 mm; (2) Partial response (PR): the sum of the diameter of target lesions is reduced by at least 30% from baseline. (3) Progressive disease (PD): the sum of the diameter of all target lesions increased at least 20% and the diameter of all lesions increased at least 5mm or found a new lesion. (4) Stable disease (SD): The decrease degree of target lesions did not reach PR level, and the increase degree did not reach PD level. Histopathological status was determined according to the 2019 WHO classification of tumors of the digestive system which based on the mitotic rate and Ki-67 index (20).

The criteria of resectability were mainly based on the counterpart in pancreatic cancer, which can be divided into resectable, borderline resectable and local advanced according to the degree of invasion of the celiac axis, hepatic artery, superior mesenteric artery, superior mesenteric vein or portal vein (21, 22). And in the current study, resection rate was defined as the number of patients undergoing surgery after neoadjuvant therapy divided by the number of patients receiving neoadjuvant therapy. R0 resection rate was defined as the number of patients with R0 resection divided by the total number of patients undergoing surgical treatment.

### 2.3 Statistical analysis

Open MetaAnalyst version 12.11.14 for windows (http://www.cebm.brown.edu/openmeta/), SPSS 26.0 and Stata was used for statistical analysis. Random effects models were used in the current study. Heterogeneity will be determined using χ^2^ test and I^2^ index. In the current study, P<0.05 and I^2^>50% were considered heterogeneity. The statistical data were presented with the proportion and 95% confidence intervals (95% CI).

## 3 Results

**Figure 1:**
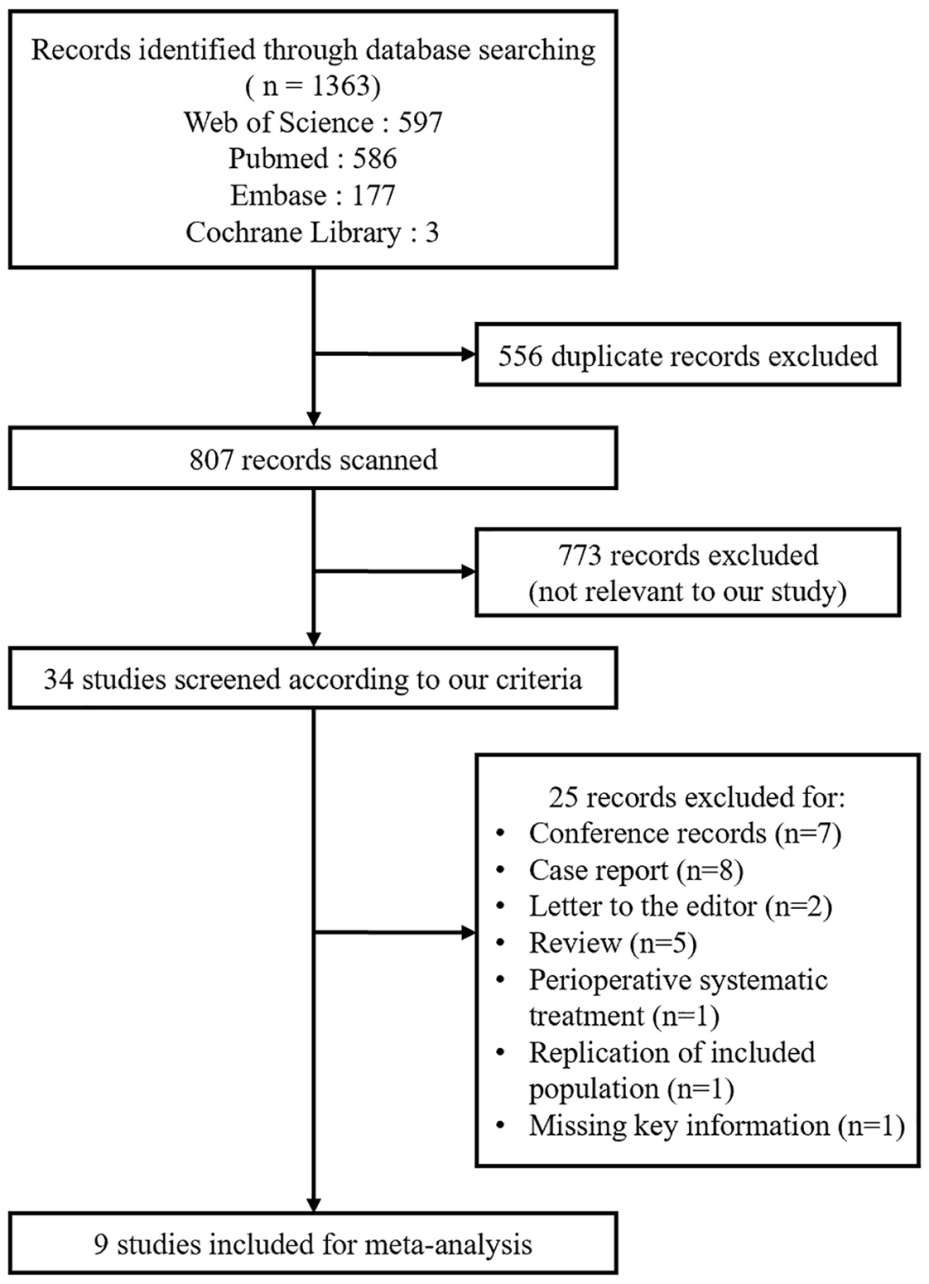
The flowchart for selecting studies.

A total of 1363 literatures were screened based on the above database and 501 duplicate articles which appeared 556 times were deleted. 773 studies were excluded because they were irrelevant to our study by reading the title and abstract. In the remaining 34 studies, there were 7 conference records, 8 case reports and 5 review excluded and 5 literatures excluded for other reasons [letter to editor (n=2), perioperative systematic treatment (n=1), replication of the study patients (n=1) and missing key information (n=1)]. Finally, we selected a total of 9 studies for meta-analysis (9, 12, 15, 23–28) (**Figure 1**).

In the selected 9 studies, there are 4 studies come from America, 3 studies published from Europe (France 1, Netherlands 1, Italy 1), 1study published from Australia and 1study from Japan. The 9 articles included a total of 468 patients from 1984 to 2019, of whom 297 patients received neoadjuvant therapy. The median sample size of each study was 29 (range: 5-112). Of the 297 cases included, 264 cases documented whether the tumor was initially resectable, and 242 cases (91.67%) were borderline resectable or unresectable tumors and 167 cases (63.26%) occurred hepatic metastases. The basic characteristics for these studies were summarized in **Table 1**.

**Table 1:** Basic characteristics of all studies included in the meta-analysis.

Author (year)	Country	Period of dataacquisition	Sample size (M/F)	Neoadjuvant therapy sample size (M/F)	Mean/median age (range/ IQR), years*	Tumor types [R/BR/U (HM)]*	Neoadjuvant therapy	Evaluation criteria for efficacy	Tumor response (CR/PR/SD/PD)	Surgery (R0/R1/R2)	Survival analysis	Median follow-up (m)
Barber (2011)	Australia	2006-2009	5 (5/0) ^♯^	4 (4/0)	63^†^(50-72)	NM	PRCRT	RECIST	0/4/0/0	1(1/0/0)	OS	15
Chenwi (2017)	USA	2000-2013	112 (NM/NM)	6 (NM/NM)	55^†^(14-70)	0/6/0 (0)	Chemotherapy (CAPTEM)	RECIST	0/2/4/0	6(4/2/0)	DFS	43
Dumont (2015)	France	1984-2013	42 (22/20)	42 (22/20)	55(25-75)	0/42/0 (0)	Chemotherapy (NM)	NM	NM	28(13/6/9)	OS	72.2
Esther (2015)	Netherlands	2000-2011	29 (14/15)	29 (14/15)	54^†^(32-81)	0/29^‡^(14)	PRRT	NM	19/ NM / NM^§^	9(6/NM/NM)	PFS	65
Jordan (2017)	USA	1998-2015	67(38/29)	27(18/9)	52 (29-74)	22/0/5 (27)	Chemotherapy (FAS)	RECIST	0/17/8/2	27(19/8/0)	OS	NM
Laura (2016)	USA	2000-2012	29 (21/8)	29 (21/8)	55 (33-81)	NM	Chemotherapy (FAS)	RECIST	0/2/26/1	14(9/5/0)	OS	88
Malcolm (2020)	USA	2009-2017	30 (19/11)	30 (19/11)	52 (49, 61)	0/10/20 (20)	Chemotherapy (CAPTEM)	RECIST	0/13/16/1	26(16/10)^£^	OS, PFS	49
Marco (2020)	Italy	2009-2018	48(31/17)	24(16/8)	53^†^( 47-75)	0/15/9 (8)	PRRT	NM	0/7/16/1	24(12/2/10)	OS	42
Yoshiki (2021)	Japan	2002-2019	106 (54/52)	106 (54/52)	57 (18-83)	0/0/106 (98)	Sunitinib	RECIST	NM	31(16/6/9)	OS, DFS	26.5

NM, not mentioned; M, male; F, female; IQR, interquartile range; R, resectable; BR, borderline resectable; U, unresectable; HM, hepatic metastases; CAPTEM, capecitabine and temozolomide; PRCRT, peptide receptor chemoradionuclide therapy; PRRT, peptide receptor radionuclide therapy; FAS, fluorouracil, doxorubicin and streptozocin; CR, complete remission; PR, partial remission; SD, stable disease; PD, progressive disease; OS, overall survival; DFS, disease free survival; PFS, Progression free survival.

*Only patients receiving neoadjuvant therapy were counted.

♯There was a case of duodenal neuroendocrine tumor included in the original study, which was not included in the current study.

†mean age.

‡R/BR or U.

§CR or PR/SD/PD.

£R0 or R1/R2.

### 3.1 Neoadjuvant therapy protocols

**Figure 2:**
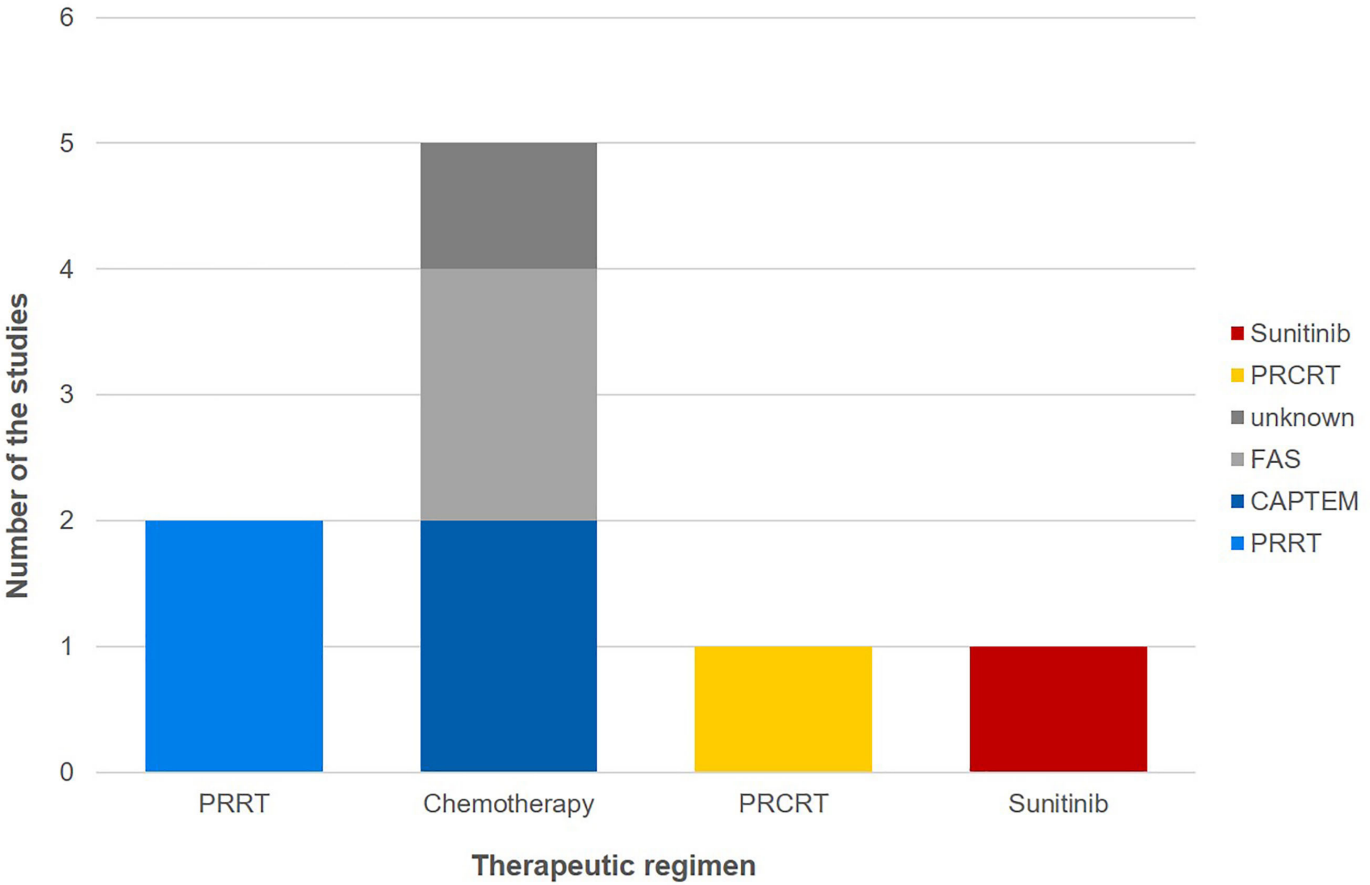
The number of studies of per neoadjuvant treatment regimen.

Among the included studies, 5 studies (55.56%) used chemotherapy as the neoadjuvant therapy regimen, 2 studies (22.22%) used PRRT, 1 study (11.11%) used peptide receptor chemoradionuclide therapy (PRCRT), and 1 study (11.11%) used sunitinib (**Figure 2**).

#### 3.1.1 Chemotherapy agents

One study did not mention the specific chemotherapy regimen they used (24). Of the four other studies using chemotherapy, two used capecitabine and temozolomide (CAPTEM) regimen and the other two used fluorouracil, doxorubicin and streptozocin (FAS) regimen. In the two studies used CAPTEM, the mean or median duration of treatment was 4-5 cycles. The dosage of capecitabine and temozolomide used in Malcolm’s study was 1500mg/m^2^ and 1500mg/m^2^ respectively (26). Among the 6 cases included in another CAPTEM regimen study, 3 cases were only treated with chemotherapy, and the other 3 cases were treated with radiotherapy on the basis of chemotherapy (23). The dose of chemotherapy drugs was not recorded in detail, but the dose of radiotherapy was 50-56 Gy and the cycle was 25-28 times. In the two studies using FAS, the dose of 5-fluorouracil was 400 mg/m^2^, doxorubicin was 40mg/m^2^, and streptozocin was 400mg/m^2^, with a median of 4 cycles for treatment (15, 25).

#### 3.1.2 PRRT, PRCRT or target therapy

The two studies using PRRT as neoadjuvant therapy regimen included a total of 53 patients, 17 (32.08%) of whom were treated with ^90^Y labeled somatostatin analogue and 36 patients (67.92%) with ^177^Lu labeled somatostatin analogue. The treatment cycles recorded in the two studies were both greater than 3 cycles, but only one study reported the specific therapeutic dose (7.4Gbq) (12, 27).

In addition, one study added 3 cycles of chemotherapy to 4 cycles of PRRT treatment. Among the 4 patients with pancreatic neuroendocrine neoplasms included in this study, the regimen of PRRT was a ^177^Lu labeled somatostatin analogue at a dose of 7-10Gbq per cycle, while the regimen of chemotherapy was 5-fluorouracil at a dose of 200mg/m^2^/24h per cycle (9).

Finally, there was a study using sunitinib as the target therapy regimen with a dose of 525mg/28d and the median treatment duration was 6 months (28).

### 3.2 Tumor response

**Figure 3:**
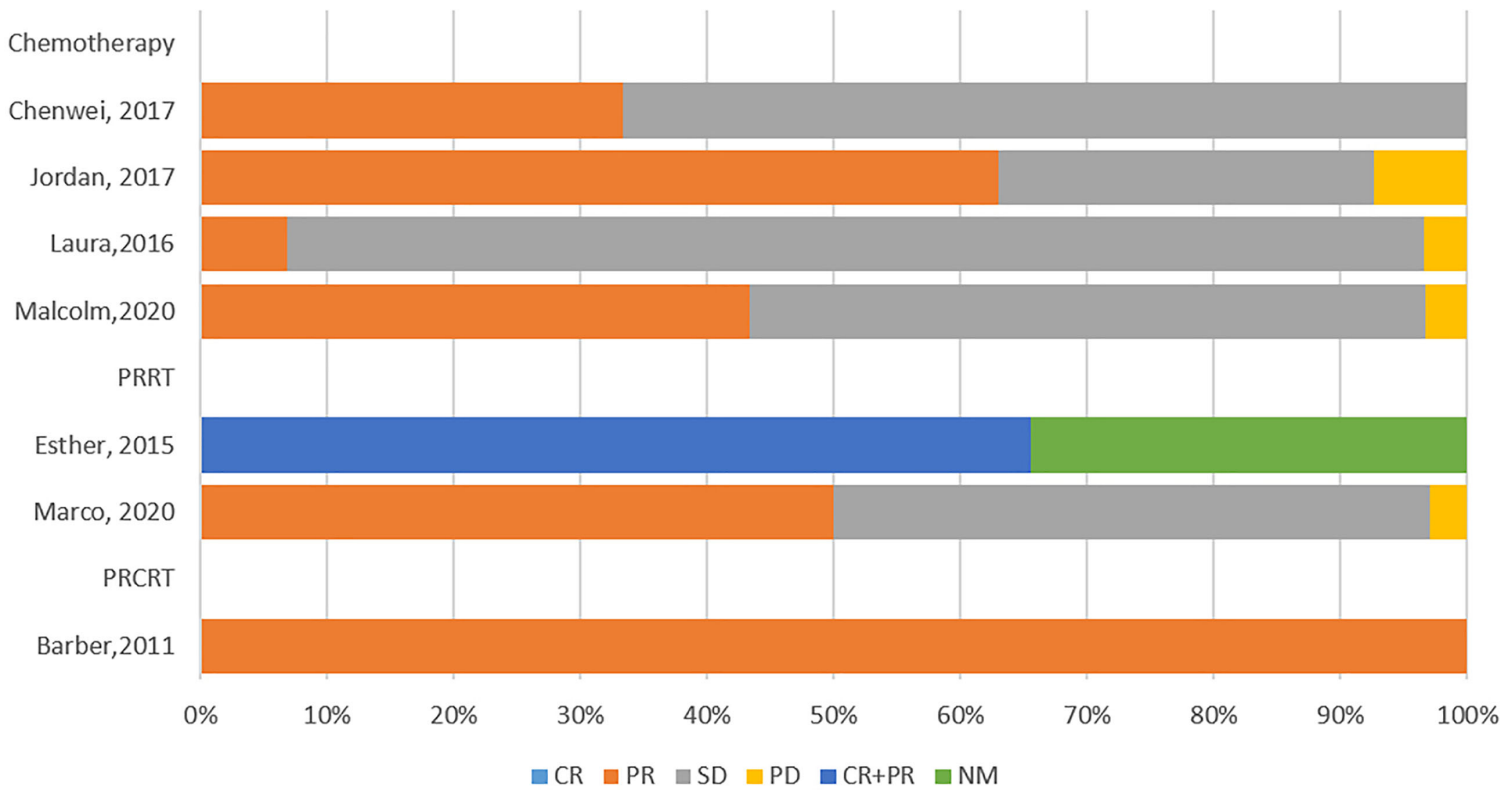
The tumor response of the included studies.

The data of tumor response was reported in 7 studies (77.78%) (**Figure 3**) and 6 of these 7studies had detailed data of tumor response which were performed further statistical analysis. RECIST criteria were used as evaluation criteria in 5 (83.33%) of these 6 studies, while in another article (16.67%) without detailed evaluation criteria, we evaluated them according to RECIST criteria based on the imaging or pathological changes.

**Figure 4:**
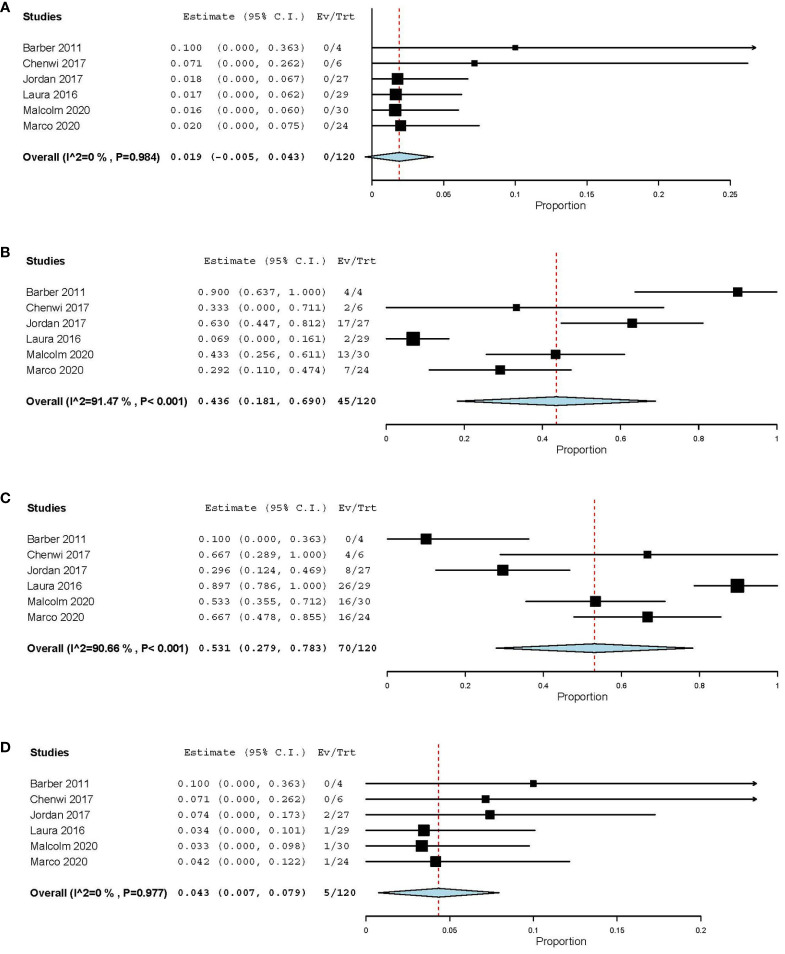
Estimate the rate of CR **(A)**, PR **(B)**, SD **(C)**, PD **(D)** of different studies.

There were 120 patients included in the 6 studies, none of the patients met the CR criteria, and the estimated CR rate was 1.9% (95%CI [-0.5, 4.3]) (**Figure 4A**). There was no significant difference in CR rates among different treatment regimens, all of which were less than 2% (P>0.05) (**Table 2**). The estimated PR rate was 43.6% (95%CI [18.1, 69.0]), and the PR rate in chemotherapy group was 36.3% (95%CI [6, 66.5]) (**Figure 4B**, **Table 2**). No bias was observed in the funnel plot of CR, PR, SD and PD (**Supplementary Figures 1A–D**). The study that used PRRT regimen only published the objective response rate (ORR), so we combined CR and PR for analysis, and the estimated ORR was 47.5% (95%CI [11.8, 83.1]) (**Table 2**). The estimated SD rate and PD rate of the 6 studies were 53.1% (95%CI [27.9, 78.3]) and 4.3% (95%CI [0.7, 7.9]), respectively (**Figures 4C, D**). In the current study, we found that there was no significant difference between patients receiving CAPTEM as neoadjuvant therapy and patients receiving FAS therapy in terms of ORR (41.67% vs 33.93%, P=0.453). Furthermore, the PRRT regimen showed a higher ORR compared to chemotherapy, however, there was also no significant difference between the two regimens (36.96% vs 49.06%, P=0.154).

**Table 2:** Estimate of tumor response after neoadjuvant therapy between different therapy regimens.

	CR	PR	SD	PD
Total [95%CI]	1.9 % [−0.5, 4.3] I^2^=0 %, P=0.984 (n=120)	43.6% [18.1, 69] I^2^=91.47 %, P<0.001 (n=120)	53.1% [29.7, 78.3] I^2^=90.66 %, P<0.001 (n=120)	4.3% [0.7, 7.9] I^2^=0 %, P=0.977 (n=120)
Chemotherapy [95%CI]	1.8 % [−0.9, 4.4] I^2^=0 %, P=0.958 (n=92)	36.3 % [6, 66.5] I^2^=91.56 %, P<0.001 (n=92)	59.8% [28.4, 91.2] I^2^=91.74 %, P<0.001 (n=92)	4.2% [0.2, 8.3] I^2^=0 %, P=0.893 (n=92)
CAPTEM [95%CI]	1.9 % [−2.4, 6.2] I^2^=0 %, P=0.58 (n=36)	41.5 % [25.5, 57.6] I^2^=0 %, P=0.638 (n=36)	55.8 % [39.6, 71.9] I^2^=0 %, P=0.531 (n=36)	3.7 % [-2.4, 9.8] I^2^=0 %, P=0.711 (n=36)
FAS [95%CI]	1.7 % [−1.6, 5.1] I^2^=0 %, P=0.972 (n=56)	34.4 % [-20.6, 89.3] I^2^=96.55 %, P<0.001 (n=56)	60 % [1.2, 118] I^2^=96.97 %, P<0.001 (n=56)	60 % [1.2, 118] I^2^=96.97 %, P<0.001 (n=56)
PRRT [95%CI]	47.5 % [11.8, 83.1] I^2^=87.59 %, P=0.005 (n=53) ^†^		Data deficient	Data deficient

†The tumor response were CR and PR.

Data deficient: The published data in the included literature were insufficient to calculate the estimate tumor response.

### 3.3 Surgical procedures

**Figure 5:**
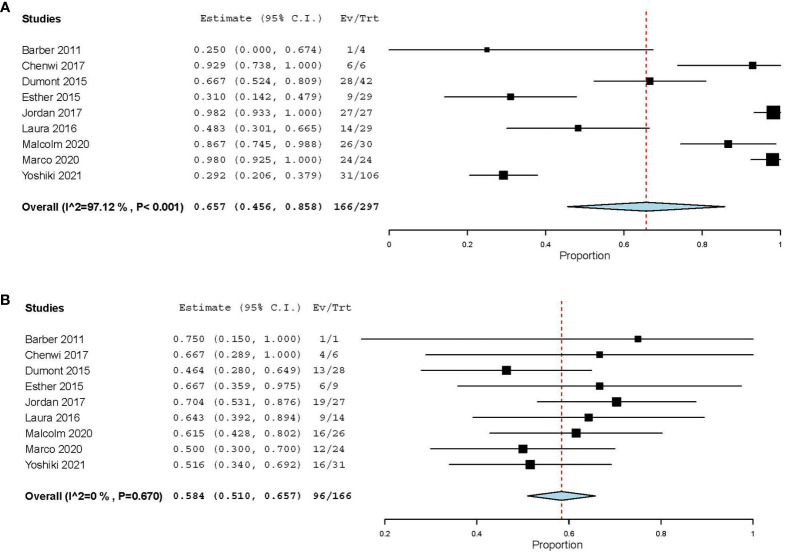
Estimate the surgical resection rate **(A)** and R0 resection rate **(B)** of different studies.

Data of surgical resection after neoadjuvant therapy were documented in all of the nine included studies. Of the 297 patients included, 166 (55.89%) received surgical resection finally, and the estimated surgical resection rate was 65.7% (95%CI [45.6, 85.8]) (**Figure 5A**). All of the nine studies reported the state of resection margin of postoperative specimens. Of the 166 patients who underwent surgical resection, 96 patients (57.83%) met the criteria for R0 resection, and its estimated resection rate was 58.4% (95%CI [51.0, 65.7]) (**Figure 5B**). No bias was observed in the funnel plot of surgical resection rate and R0 resection rate (**Supplementary Figures 1E, F**).In the four included studies that used chemotherapy, we found that there was no significant difference in R0 resection rate between patients receiving CAPTEM and patients receiving FAS therapy (62.50% vs 68.30%, P=0.605). **Table 3** summarized the difference of estimate resection rate and estimate R0 resection rate between chemotherapy regimen and PRRT regimen. We could find that the study used CAPTEM had the highest resection rate (88.5%, 95% CI [78.2, 98.7]), followed by FAS (74%, 95%CI [25.1, 123]), while PRRT had the lowest resection rate (65%, 95%CI [-0.6, 130.6]).

**Table 3:** Estimate of surgical resection rate and R0 resection rate after neoadjuvant therapy between different therapy regimens.

	Resection rate	R0 resected
Total [95%CI]	65.7% [45.6, 85.8] I^2^=97.12 %, P<0.001 (n=297)	58.4% [51, 65.7] I^2^=0 %, P=0.67 (n=166)
Chemotherapy [95%CI]	79.3% [61.9, 96.7] I^2^=90.29 %, P<0.001 (n=134)	61% [51.6, 70.3] I^2^=0 %, P=0.451 (n=101)
CAPTEM [95%CI]	88.5 % [78.2, 98.7] I^2^=0 %, P=0.592 (n=36)	Data deficient
FAS [95%CI]	74 % [25.1, 123] I^2^=96.3 %, P<0.001 (n=56)	68.4 % [54.2, 82.6] I^2^=0 %, P=0.695 (n=41)
PRRT [95%CI]	65 % [-0.6, 130.6] I^2^=98.18 %, P<0.001 (n=53)	54.9 % [38.2, 71.7] I^2^=0 %, P=0.374 (n=33)

Four studies, including 74 patients who underwent surgery, reported detailed information about the procedure. 43 (58.11%) patients received distal pancreatectomy, 17 (22.97%) received pancreaticoduodenectomy, 6 (8.11%) patients only received hepatectomy for the liver metastases and the procedures of the remaining 8 (10.81%) patients were not clearly described. Due to portal vein invasion and liver metastasis, partial portal vein resection and reconstruction were performed in 4 (5.41%) patients. Vascular resection was also performed in other three patients, but the specific vessels resected were not described amply. Hepatic segmentectomy or combined radiofrequency ablation was performed in 24 (32.43%) patients. 39 (52.70%) of the 74 patients achieved R0 resection. Varying degrees complications occurred in 21 (28.38%) patients during hospitalization, with pancreatic fistula being the most common complication, which occurred in 9 (33.33%) patients. Among the four studies, three reported the duration of surgery and the amount of blood loss. The mean of the median duration in per study was 405.83 minutes (range: 195-629minutes), and the mean amount of blood loss was 786ml (range: 65-8000ml).

### 3.4 Histological response

Five (5/9, 55.56%) studies described the pathological grade of postoperative specimens. Of these studies, 102 patients were eventually treated with surgery, 30 (29.41%) patients were pathologically diagnosed with NET G1, 53 (51.96%) were diagnosed with NET G2 and 6 (5.88%) patients were diagnosed with NET G3. What’s more, 1 (0.98%) patient was eventually diagnosed with NEC and the remaining 12 (11.76%) patients did not obtain the definite pathological stage eventually. Unfortunately, due to the lack of pathology details, the role of neoadjuvant therapy in tumor pathological response cannot be clearly evaluated.

### 3.5 Survival analysis

Our study further analyzed the survival time after neoadjuvant therapy. Median follow-up time was documented in 8 studies (88.89%) of the included studies. The mean of the median follow-up time was 50.9 months (range:15-88 months). And we found 3 studies documented the overall survival time for the patients who received and did not receive surgical resection. Among the three studies, FAS was used as the neoadjuvant therapy in 2 studies and sunitinib was used in 1 study. **Table 4** shows the details of the patients’ prognosis. It is clear that the prognosis of patients who underwent surgery after neoadjuvant therapy was significantly better than those who did not undergo surgery.

**Table 4:** Overall survival time between surgery group and no surgery group.

	Neoadjuvant therapy	Median overall survival time (95% CI) (months)	
		Surgery	No surgery
Jordan, 2017	FAS	108.2 (73.2-143.2)	59.6 (42.5-76.8)
Laura, 2016	FAS	112 (104-120)	41 (16-66)
Yoshiki, 2021	Sunitinib	>72^*^	36.7

*By the end of follow-up time, the median survival time had not been reached.

### 3.6 Assessment of sensitivity analysis and publication bias

Begg’s test and Egger’s linear regression test were used to assess whether there was potential publication bias in this meta-analysis. The results showed that no apparent publication bias for the analysis was found between PR, SD and R0 resection rate (**Supplementary Figures 2B, C, F**, **3B, C, F**). However, significant publication bias was found CR, PD and surgical resection rate (**Supplementary Figures 2A, D, E** and **Supplementary Figures 3A, D, E**). And the P value of Begg’s test and Egger’s test was listed in **Supplementary Table 3**. Sensitivity analysis was performed to evaluate the stability of tumor response, surgical resection rate and R0 resection rate after neoadjuvant therapy. After removing any of the studies, the results did not exceed the 95% CI range of the pooled results (**Supplementary Figures 4A–F**).

## 4 Discussion

In the past, cytotoxic chemotherapy was only used for pNENs that was difficult to resect surgically or had distant metastases, and surgery was still the first option if it is technically possible. Although in patients who were difficult to resect radically, tumor reduction surgery also seemed to be beneficial to patient survival (26). Neoadjuvant therapy offers the theoretical benefit of “downstaging” in patients who cannot be surgically resected because of vascular involvement or distant metastases and potentially reduces the risk of recurrence. However, unlike pancreatic cancer, where the positive role of neoadjuvant therapy had been widely reported, the role of neoadjuvant therapy in pNENs was still unknown (29–31). The National Comprehensive Cancer Network (NCCN) guidelines updated to recommend chemotherapy, targeted therapy, PRRT and other neoadjuvant therapies for unresectable, well-differentiated G3 stage pNET with high tumor burden and also recommended for resectable lesions with unfavorable biology (e.g. Ki67>55%, rapid growth, negative SSR-based PET imaging) to achieve the best clinical efficacy (32). The North American Neuroendocrine Tumor Society (NANET) consensus also indicate that neoadjuvant therapy may be a downstaging treatment option for pNENs with locally advanced or distant metastasis, especially prior to tumor reduction surgery (33). However, the European Neuroendocrine Tumor Society (ENETS) guidelines did not recommend surgery for high-grade pNENs with locally advanced and distant metastasis diseases, and noted that the role of neoadjuvant therapy is unclear (10). To date, the choice of treatment strategy for local advanced or distant metastatic pNENs was still lack widely consensus, the effect of neoadjuvant therapy for unresectable or borderline resectable pNENs was still not clear, and lack systematic evaluation, and the pros and cons of each regimens also lack the systematic comparison.

Currently, some studies have revealed the positive effect of drug therapy on tumor reduction of pNENs, which are consistent with our results. Chemotherapy, PRRT and targeted therapy are the most studied neoadjuvant therapies for pNENs. PRRT therapy is widely used due to well-differentiated pNETs expressed somatostatin receptors (14, 34, 35). Well-differentiated pNENs often express many characteristic biomarkers, such as Chromogranin A (CgA) and somatostatin receptor subtype 2 (SST2) (36, 37). SST2 can be highly bound to somatostatin analogs (SAAs), such as octreotide. SAAs labeled with radionuclides have both diagnostic and therapeutic effects in pNENs. In addition to tracing, it can also damage the DNA of recipient cells, thereby inhibiting the proliferation of pNENs (38). Daniel and Samer et al. have reported successful shrinkage and surgical treatment of advanced pNENs after using PRRT regimens (14, 35). Because most studies were case reports, the evidence for the role of PRRT in the neoadjuvant therapy of pNENs was not strong enough. At the same time, there was a lack of prognostic information in their study, so it was not clear whether the application of PRRT was beneficial to the prognosis of patients. In 2019, Swayamjeet et al. published a systematic review and meta-analysis comparing the effects of PRRT and everolimus in advanced pNENs (39). The study found that PRRT was superior to everolimus in terms of objective response rate, disease control rate, and survival time (39). Meanwhile, PRRT therapy has better safety in terms of hematological toxicity and nephrotoxicity (39). In the literatures included in the current study (2 studies), the objective response rate of PRRT was 49.1%, which was similar to the study of Swayamjeet (R=47%).In our study, 62.3% of patients could be restored to surgery, and the median follow-up time of the two studies was 53.5 months, which was significantly better than the patients treated only with PRRT in the study of Swayamjeet (25.7 months) (39). The main reason for this difference may be the different subjects included in the study. Among the subjects included in our study, PRRT was only used as neoadjuvant therapy before surgery. Swayamjeet et al. did not specify the subjects included in their study, which mainly observed the response of PRRT treatment in patients who could not receive surgical treatment, and the included subjects were highly heterogeneous. Since not all neuroendocrine tumors were highly expressed somatostatin receptors, some pNENs showed low reactivity to somatostatin, which may lead to poor reaction of PRRT (8). Therefore, chemotherapy is also widely used in pNENs, especially for poorly differentiated advanced tumors. Furthermore, pNENs are generally considered to be sensitive to chemotherapy, and chemotherapy was the first-line treatment for pNENs which aimed to reduce tumor cells (8). However, chemotherapy is more toxic than PRRT (40). In well-differentiated G3 stage pancreatic neuroendocrine tumors, alkylating agents are widely used, such as CAPTEM regimen containing temozolomide and FAS regimen containing streptomycin (8, 41, 42). Platinum agents were more commonly used in pNEC (8, 43). In a retrospective study of patients with G3 stage pancreatic neuroendocrine tumors and pNEC, Nitya and colleagues found that there was no difference in the response rates of alkylating agents and platinum agents between the two groups, while platinum agents were significantly superior to alkylating agents in pNEC (43). Since there were few studies on the efficacy comparison between different chemotherapy regimens, the advantages and disadvantages of different chemotherapy regimens cannot be analyzed. Among the literatures on chemotherapy included in the current study, there were two literatures on CAPTEM and the other two literatures on FAS (15, 23, 25, 26). Combined with the above analysis, there was no significant difference in R0 resection rate (P=0.605) and objective response rate (P=0.154) between the two chemotherapy regimens. In the current study, the objective response rate of chemotherapy was 36.96%, which was not significantly different from PRRT (R=49.06%, P=0.129).

Nowadays, the genetic changes in PNENs have been widely revealed. The most common alterations found in pNENs are in various genes in the MEN1, DAXX/ATRX and mTOR pathways (44). Targeted drugs, such as sunitinib, are increasingly being used in clinical trials. Sunitinib is an anti-angiogenic drug. It is a multi-target tyrosine kinase inhibitor that can inhibit VEGFR and PDGFR, etc (45, 46). It can play an anti-tumor angiogenesis role in pNENs. Many clinical trials have shown that sunitinib had a good objective response rate for advanced pNENs, prolonged patient survival, and showed good safety with few side effects, however, they published that the ORR of pNENs treated with sunitinib was about 9.3%-17%, which was significantly lower than chemotherapy (36.96%) and PRRT (49.06%) included in the current study (47–50). However, studies on the role of targeted drugs in neoadjuvant therapy of pNENs are very rare. And there was only 1 study about sunitinib as the neoadjuvant therapy regimen included in our study, and the side effects of targeted therapy were not detailed description in this study (28). Unfortunately, in this study, 29.25% (31/106) of patients successfully completed “down-staging” therapy and underwent surgery after sunitinib, but the objective response rate of sunitinib was not described in this study.

Our study is the first systematic review and meta-analysis of neoadjuvant therapy for pNENs related to tumor response, surgical rate, R0 resection rate, and prognosis. In our study, we found that there were 46.54% (74/159) patients reached partial response, 44.23% (70/159) patients had a stable disease, and only 3.14% (5/159) patients experienced progressive disease. Meanwhile, 67.30% (107/159) of these patients were given the chance of surgery again, and the R0 resection rate was greater than 47.66% (51/107). Therefore, neoadjuvant therapy could effectively limit the progression of neuroendocrine tumors and improve the probability of surgical resection.

However, there are still many deficiencies in our research: (1) The literatures included in this study were all retrospective studies and the sample size included in each study was generally small, which may lead to the existence of bias in the process of data collection, which also leads to the fact that this study has little guiding significance for existing clinical problems. (2) The criteria for tumor resectability and the indications for neoadjuvant therapy were not clearly explained among different studies. And these factors may be the main sources of heterogeneity. (3) Since there was no control group in each study, we can only observe the ratio within each study, and it is difficult to judge the advantages and disadvantages of different studies, as well as different treatment strategies.

In conclusion, neoadjuvant therapies, such as chemotherapy and PRRT, could reduce the volume and stage of some borderline resectable or unresectable pNENs, and gave some patients the chance of radical resection. However, the current study was not able to identify differences in efficacy between different treatment regimens. Next, it is necessary to conduct prospective clinical studies on the role of neoadjuvant therapy in pNENs, which should include clear definitions and criteria for tumor resectability.

## Data availability statement

The original contributions presented in the study are included in the article/**Supplementary Material**. Further inquiries can be directed to the corresponding author.

## Author contributions

The author contributions are as follows: ZF and HZ conceived the project. YL and FZ wrote the manuscript. ZF and YL searched the literature together. JY and MS completed the data extraction together. YM and SL participated in the data analysis and visualization. All authors contributed to the article and approved the submitted version.

## Funding

This work was supported by the National Natural Science Foundation of China (81702365, 81972274), Shandong Provincial Natural Science Foundation (ZR2021LSW004, ZR2017MH090), Clinical Research Center of Shandong University (2020SDUCRCC016), Taishan Scholars Program for Young Expert of Shandong Province (tsqn202103172).

## Conflict of interest

The authors declare that the research was conducted in the absence of any commercial or financial relationships that could be construed as a potential conflict of interest.

## Publisher’s note

All claims expressed in this article are solely those of the authors and do not necessarily represent those of their affiliated organizations, or those of the publisher, the editors and the reviewers. Any product that may be evaluated in this article, or claim that may be made by its manufacturer, is not guaranteed or endorsed by the publisher.
